# Distance measurements between 5 nanometer diamonds – single particle magnetic resonance or optical super-resolution imaging?†‡

**DOI:** 10.1039/d2na00815g

**Published:** 2023-01-24

**Authors:** Dorothea Pinotsi, Rui Tian, Pratyush Anand, Koichiro Miyanishi, Jens M. Boss, Kevin Kai Chang, Pol Welter, Frederick T.-K. So, Daiki Terada, Ryuji Igarashi, Masahiro Shirakawa, Christian L. Degen, Takuya F. Segawa

**Affiliations:** a Scientific Center for Optical and Electron Microscopy (ScopeM) ETH Zurich 8093 Zürich Switzerland; b Laboratory for Solid State Physics ETH Zurich 8093 Zürich Switzerland; c High-Field MR Center, Max Planck Institute for Biological Cybernetics Tübingen Germany; d Graduate School of Engineering Science, Osaka University Toyonaka Osaka 560-8531 Japan; e Center for Quantum Information and Quantum Biology, Osaka University Osaka 560-8531 Japan; f Neurocritical Care Unit, Department of Neurosurgery and Institute of Intensive Care Medicine, University Hospital Zurich 8091 Zürich Switzerland; g Department of Molecular Engineering, Graduate School of Engineering, Kyoto University Nishikyo-Ku Kyoto 615-8510 Japan; h Institute for Quantum Life Science, National Institutes for Quantum Science and Technology Anagawa 4-9-1, Inage-Ku Chiba 263-8555 Japan; i Institute of Chemical Research, Kyoto University Uji Kyoto 610-0011 Japan; j Laboratory of Physical Chemistry ETH Zurich 8093 Zürich Switzerland segawat@ethz.ch

## Abstract

5 nanometer sized detonation nanodiamonds (DNDs) are studied as potential single-particle labels for distance measurements in biomolecules. Nitrogen-vacancy (NV) defects in the crystal lattice can be addressed through their fluorescence and optically-detected magnetic resonance (ODMR) of a single particle can be recorded. To achieve single-particle distance measurements, we propose two complementary approaches based on spin–spin coupling or optical super-resolution imaging. As a first approach, we try to measure the mutual magnetic dipole–dipole coupling between two NV centers in close DNDs using a pulse ODMR sequence (DEER). The electron spin coherence time, a key parameter to reach long distance DEER measurements, was prolonged using dynamical decoupling reaching *T*_2,DD_ ≈ 20 μs, extending the Hahn echo decay time *T*_2_ by one order of magnitude. Nevertheless, an inter-particle NV–NV dipole coupling could not be measured. As a second approach, we successfully localize the NV centers in DNDs using STORM super-resolution imaging, achieving a localization precision of down to 15 nm, enabling optical nanometer-scale single-particle distance measurements.

## Abstract 2

5 nanometer sized detonation nanodiamonds (DNDs) are studied as potential single-particle labels for distance measurements in biomolecules.

## Graphical abstract

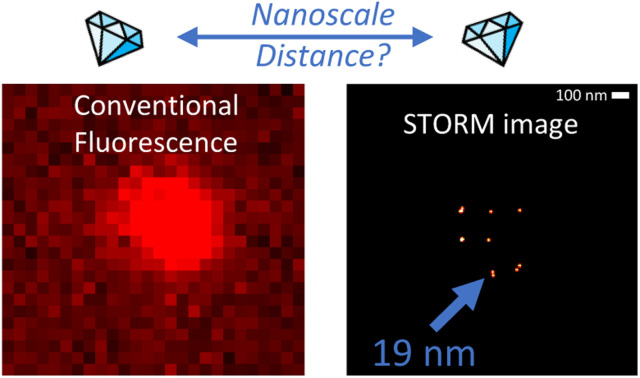

## Introduction

One of the key challenges of nanoscience and -technology is the visualization of nanoparticles: the smaller the particles, the bigger the challenge. In this work, we propose two complementary optical experiments to measure distances between 5 nanometer-sized detonation nanodiamonds (DNDs). DNDs are the smallest class of nanodiamonds, which can be produced in large quantities.^1^ They show a spherical shape and a rather uniform size distribution of 4–5 nm. Imaging of these particles is of particular interest, since they have for example been successfully used as a drug delivery system in humans.^2^ While larger nanodiamond particles can be optically detected thanks to their high refractive index *via* scattering^3^ or diffraction experiments,^4^ a single-digit (<10 nm) nanodiamond remains invisible with these techniques. Alternatively, a special optically active crystal defect can be used to visualize a single particle with the help of a highly sensitive fluorescence microscope. The defect of interest is the nitrogen–vacancy (NV) color center, where a nitrogen impurity atom replaces a carbon atom in the diamond lattice and one neighboring lattice site remains empty (vacancy).^5,6^ Fluorescence from NV centers inside DNDs was observed^7,8^ and even signals from individual NV centers in single DNDs detected.^9^ However, for such a conventional fluorescence detection, the spatial resolution is diffraction limited and a nanoscale distance between two close DNDs remains blurred because it is far below the diffraction limit. If such a measurement could become accessible, by attaching nanometer-sized DNDs as fluorescent labels to biomolecules using controlled surface chemical modification,^10^ structural changes on a single molecule-level could be targeted and visualized.

**Fig. 1:**
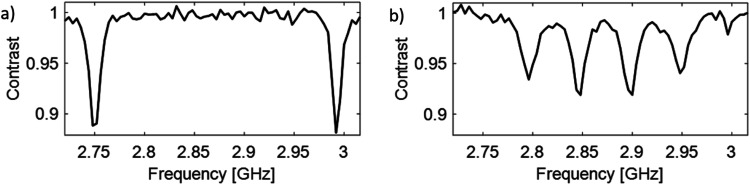
ODMR spectra of NV^−^ centers in DNDs under a weak external magnetic field (<10 mT). (a) ODMR spectrum for a single NV^−^ center in a DND, showing two ODMR resonances *m*_S_ = −1 ←→ *m*_S_ = 0 and *m*_S_ = +1 ←→ *m*_S_ = 0, centered around the zero-field splitting *D* = 2.87 GHz. (b) ODMR spectrum for two NV^−^ center in two DNDs, showing four ODMR resonances due to their different spatial orientation of the N–V axis with respect to the external magnetic field. Spots with such a signature were chosen to perform relaxation and DEER experiments.

Our approaches for distance measurements between the 5 nm DNDs are inspired by two physicochemical techniques. The first approach originates from the field of magnetic resonance, namely electron paramagnetic resonance (EPR) spectroscopy. Using so-called “spin labels” (stable radicals), distances between two unpaired electron spins can be inferred from spectroscopic measurements of their mutual dipole–dipole coupling – a technique known as double electron–electron resonance (DEER) spectroscopy.^11^ DEER spectroscopy is an ensemble technique, which usually needs about 10^15^ spins to detect a signal at cryogenic temperatures. DEER measurements were successfully demonstrated between single NV centers in an ultra-pure bulk diamond crystal at room temperature.^12^ More recently, a single electron spin located on a fullerene-encapsulated nitrogen atom (^14^N@C_60_) was detected using DEER from an NV center on a diamond nanopillar at 4.7 K.^13^ This incredible gain in sensitivity is achieved through optical detection and polarization of the unpaired electron spins in the negatively-charged NV^−^ centers (“optically detected magnetic resonance” – ODMR), which enables EPR spectroscopy of a single NV center.^14,15^ We show that the prerequisites for DEER measurements between two close DNDs, each containing an NV^−^ center, are fulfilled, but the experimental realization remains challenging. Our second approach is a purely optical one and uses stochastic optical reconstruction microscopy (STORM)^16,17^ a fluorescence based technique that overcomes the diffraction limit, to obtain super-resolved fluorescence images. This technique was successfully applied to image multiple NV centers inside *ca.* 75 nm diamonds.^18^ Further, it was shown for NV centers in bulk diamond that STORM super-resolution can be combined with the readout of magnetic resonance spectra.^19^ Herein, we show that this method can be applied to measure distances between individual 5 nm DNDs containing NV centers, rather than distances within one larger diamond particle. These two methods complement the ODMR-based “deterministic emitter switch microscopy” (DESM) technique, which can be implemented on a confocal or wide-field ODMR microscope.^20^ By applying a microwave irradiation resonant to an ODMR transition of an NV^−^ center in one nanodiamond (see Fig. 1), the fluorescence can be selectively reduced during image acquisition, which is the basis for the reconstruction of a super-resolved image similar to STORM. Using this ODMR-based DESM approach, we have recently measured distances between NV^−^ centers in 5 nm DNDs as small as 33 nm on a wide-field microscope.^21^ An overview of the three different nanoscale distance measurement methods is given in Table 1.

**Table 1:** Comparison of optical nanoscale distance measurement methods between DNDs containing NV centers

Technique	Measurement principle	Experimental setup	Required properties of NV center	Accessible distance range	Advantages	Disadvantages
STORM (stochastic optical reconstruction microscopy)	*Super-resolution* fluorescence imaging based on stochastic photo-switching (blinking) leads to localization of single emitters with nanometer precision	Wide-field fluorescence microscope	Fluorescence blinking	Min. distance: *ca.* 15 nm, max. distance: only limited by the FOV	Can be carried out on a commercial fluorescence microscope; purely optical technique; does not rely on spin properties of NV centers; high throughput – positions of all NV centers in the FOV measured at once	Min. distance limited by thermal drift and number of photons detected

ODMR-based DESM (deterministic emitter switch microscopy)	*Super-resolution* fluorescence imaging based on deterministic photo-switching using the ODMR effect leads to localization of single emitters	Wide-field or confocal fluorescence microscope for ODMR (incl. magnet, CW MW)	Photostability; large ODMR contrast	Min. distance: *ca.* 15 nm,^21^ max. distance: only limited by the FOV	Does not rely on electron spin coherence time *T*_2_	Min. distance limited by thermal drift; CW ODMR setup (magnet and CW MW) needed; low throughput – for each NV orientation (ODMR frequency) an individual image has to be recorded

DEER (double electron–electron resonance)	*Magnetic resonance* spectroscopic determination of the magnetic dipole–dipole coupling between two electron spins	Confocal fluorescence microscope for ODMR (incl. magnet and pulsed MW)	Photostability; large ODMR contrast; long electron spin coherence time *T*_2_	Experimentally not realized, estimated max. distance (for *T*_2,DD_ = 20 μs): *ca.* 10 nm (depending on orientation of N–V axis)	Measurement of dipole–dipole coupling *ω*_DD_ is independent of thermal drifts	Distance must be derived from the dipole–dipole coupling *ω*_DD_ (incl. Orientation of N–V axis); strong selection criteria on spin properties of NV centers

The negatively-charged NV^−^ center is EPR active, since it has an electron spin *S* = 1 with two unpaired electrons in degenerate molecular orbitals.^5,6^ EPR room-temperature coherence times under dynamical decoupling *T*_2,DD_ of >60 μs in ultrapure nanodiamonds^22^ and even up to milliseconds in engineered bulk diamonds^23^ open the way for versatile pulse magnetic resonance experiments, such as the spin echo or the DEER sequence, where the spin state can be controlled to a high degree.

The experiment workflow is as follows: NV^−^ centers are first localized with a confocal fluorescence microscope. In a second step, the ODMR spectrum is recorded at the location of the maximum fluorescence intensity by applying laser (for optical polarization/detection) and resonant microwave excitation (for electron spin manipulation). The intensity of the fluorescence signal depends on the spin state of the NV^−^ electron: while electrons in the spin state *m*_*S*_ = 0 emit bright fluorescence, the fluorescence of electrons in the spin state *m*_*S*_ = ±1 appears about 30% darker.^6^ This difference is called the ODMR contrast and is the key to the optical detection of the electron spin states in NV^−^ centers. To achieve a decent signal-to-noise ratio, ODMR experiments are repeated many times over minutes or even hours.

**Fig. 2:**
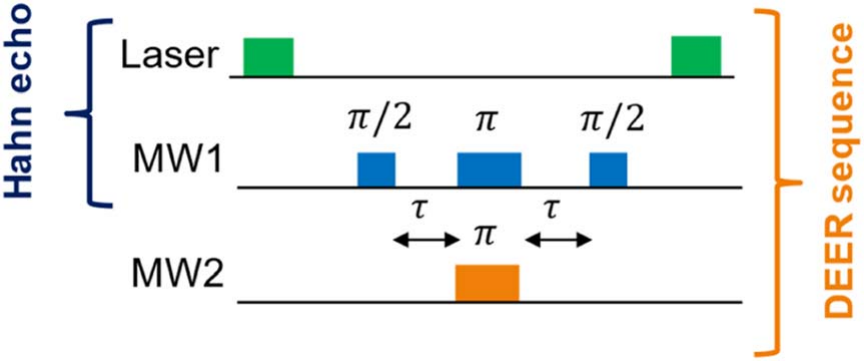
Hahn echo^24^ and DEER pulse sequence in ODMR spectroscopy for NV^−^ centers in diamonds. The green laser pulses (around 10 μs) polarize the EPR transition for the upcoming MW pulse sequence and read out the electron spin state at the end of each MW pulse sequence. The blue MW pulses (usually tens of nanoseconds) on the channel “MW1” are resonant with an EPR transition of the first NV^−^ center (NV1). The pulses with the flip angles π/2 and π, separated by the delays τ, form the Hahn echo, while the last π/2 pulse is necessary to flip back the magnetization to a optically-detectable spin state.^25^ The orange π-pulse is resonant with an EPR transition of the second NV^−^ center (NV2) and timed synchronously to the π-pulse of the Hahn echo on channel MW1. This refocuses exclusively the NV1–NV2 dipole–dipole coupling.^26^

The DEER pulse sequence (see Fig. 2) consists of two elements: a Hahn echo with blue microwave (MW) pulses on the first NV center (“NV1”) and a single π-pulse (orange pulse) on the second NV center (“NV2”). This scheme requires that the two NV^−^ centers of interest can be spectroscopically discriminated, *i.e.*, they need a distinguishable ODMR resonance frequency.

This is achieved by applying a small magnetic field (<10 mT) using a permanent dipole magnet to add a small Zeeman contribution to the zero-field splitting.^6^ The EPR resonance frequency depends on the orientation of the NV^−^ centers inside the nanodiamonds^20,21^ through the zero-field splitting interaction, whose principal axis is aligned with the N–V direction in the diamond lattice. By increasing the delay time *τ* in the DEER pulse sequence, a time trace is obtained, which will decay with the coherence time *T*_2_ and oscillate with the mutual dipole–dipole coupling *ω*_DD_ between NV1 and NV2.

The dipole–dipole coupling *ω*_DD_ is inversely proportional to the cube of the inter-spin distance *r*^3^ and depends on the angles between the spins and the magnetic field axis. In EPR spectroscopy of large ensembles, the distance *r* can be extracted by integrating over all possible spin orientations.^11^ This is not possible for single-particle distance experiments between two NV^−^ centers. Moreover, the zero-field splitting of NV^−^ centers of *D* = 2.87 GHz is much larger than the electron Zeeman splitting *γB* < 280 MHz (for *B* < 10 mT, with *γ* = 28 GHz T^−1^ being the electron gyromagnetic factor). Therefore, the NV^−^ spins will not be quantized along the external magnetic field, as usually for DEER in high-field EPR spectroscopy, which will further increase the degrees of freedom. For a DEER measurement between two NV^−^ centers in different DNDs, this will lead to a distance range rather than an exact distance *r*. The maximum accessible distance *r*_max_ is related to the electron spin coherence time *T*_2_ of the NV^−^ center, which defines the maximum observation time of the DEER signal (*i.e.*, the longest *τ* delay). The maximum distance that can be estimated is about^11^1
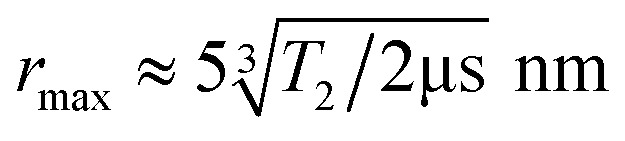


The equation highlights the challenging dependence on the cubic root of *T*_2_: to double the maximum achievable distance *r*_max_, the coherence time *T*_2_ must be prolonged by a factor of eight.

The basis of STORM super-resolution imaging, our second approach towards distance measurements, is the “blinking” of a fluorophore, *i.e.*, a stochastic switching between fluorescence “on” and “off” states. The stochastic and sparse switching of the fluorophores allows localization of individual fluorescent spots with a resolution that is significantly finer than the diffraction limit of *ca. λ*/2 with *λ* being the wavelength of the emitted light. A highly sensitive camera in a wide-field fluorescence microscope records a series of images, which are processed in an image analysis step. This leads to a reconstructed image, where the center of a two-dimensional Gaussian becomes the localization of an individual spot. The localization precision scales with 1/√*N*, where *N* is the number of collected photons: the more photons collected, the higher will be the spatial resolution.^17,27^ The reason for a limited experimental spatial resolution is bleaching, a laser-induced structural change of the fluorophore, which leads to a permanent disappearance of its fluorescence. Another key requirement is a very high microscope stability, since thermal motion in the order of nanometers might introduce drifts over the total acquisition time of a STORM sequence.

NV centers in diamonds have been praised as stable fluorescence emitters, which do not show any blinking or bleaching. However, NV^−^ centers close to the diamond surface (within several nanometers), especially in nanodiamonds, are the important exception to this rule.^9^ The blinking in the case of the crystal defect is a charge effect: the negatively-charged NV^−^ center is photoionized into its neutral state NV^0^, where this effect is reversible.^28^ Both charge states of the NV center are fluorescent, but the emission from NV^0^ is shifted to shorter wavelengths.^29^ A recent study, spectrally discriminating the NV^−^ and NV^0^ charge states, showed that the equilibrium shifts towards NV^0^ for very small (<10 nm) nanodiamonds.^30^ For given spectral filter settings of the microscope, this can lead to observation of blinking (switching “on” and “off” between dark and bright states).

## Results and discussion

### DEER

**Fig. 3:**
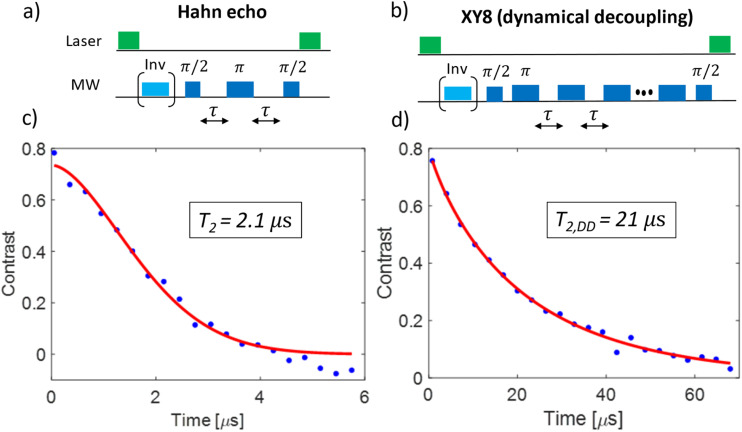
Coherence time *T*_2_ measurements of a single NV^−^ center in a 5 nm DND under a Hahn echo and under dynamical decoupling (XY8 sequence) with a maximum of 176 π-pulses. (a) and (b) show the pulse sequences for the Hahn echo and the dynamical decoupling sequence, respectively. The light blue pulses inside brackets depict the adiabatic inversion pulses, which are applied for every second run (see raw data in ESI‡). The inverted signal (when blue pulse applied) is subtracted from the normal one to correct for the background signal (“phase cycling”). (c) and (d) show the experimental data (blue dots) and the fits (red line) for the Hahn echo and the dynamical decoupling sequence, respectively. While the Hahn sequence achieves *T*_2_ = 2.1 ± 0.2 μs, the dynamical decoupling sequence prolongs the coherence time by factor of 10 reaching *T*_2,DD_ = 21 ± 2 μs. This corresponds to more than doubling of the maximum DEER distance *r*_max_ (see eqn (1)). The duration of the π-pulse was 38 ns (Rabi oscillation, see ESI‡), the inter-pulse delay during the dynamical decoupling was to *τ* = 62 ns. The free induction decay (FID) time of this NV^−^ center was *T*_2_* = 30 ns (see ESI‡).

An initial ODMR spectrum without a magnetic field was recorded. A typical signal at the characteristic zero-field splitting of *D* = 2.87 GHz enabled a simple control, whether the origin of the fluorescence was the NV^−^ defect. Then, a second ODMR spectrum with an applied magnetic field (<10 mT) was recorded. A spectrum with two lines indicated a single NV^−^ center, while four lines (two transitions *m*_*S*_ = 0 ↔ *m*_*S*_ = +1 and *m*_*S*_ = 0 ↔ *m*_*S*_ = −1 for each NV^−^ center) indicated the presence of two NV^−^ centers with different orientations within the confocal spot (Fig. 1(b)). In case of overlapping lines, the magnet position was modified to achieve a clear separation of the lines for the DEER experiment. The ODMR contrast was in the best cases around 10% (see Fig. 1). This is lower compared to the ODMR contrast of NV^−^ centers in bulk diamonds with around 30%. The decrease in ODMR contrast is associated with remaining fluorescent surface groups on DNDs (*e.g.*, sp^2^ carbon) outshining the NV^−^ fluorescence and a fast exchange between NV^−^ and NV^0^, where only the former charge state shows an ODMR effect. EPR pulses for coherent excitation (π/2-pulse) and refocusing or inversion (π-pulse) were calibrated by recording a Rabi oscillation on the ODMR resonance frequency (see Fig. S3 in ESI‡). The channel MW1 (see Fig. 2, blue pulses) was set for the transition with the highest ODMR contrast to maximize detection sensitivity. Usual coherence times were around *T*_2_ ≈ 1 μs or shorter, while the longest values were *T*_2_ ≈ 4 μs as previously reported.^21^ By using our estimate for the achievable maximum distance shown in eqn (1), we obtain a maximum DEER distance of *r*_max_ ≈ 6 nm for *T*_2_ ≈ 4 μs. In other words, depending on the position of the NV^−^ center inside a 5 nm DND particle, even adjacent diamond particles would not guarantee an observable dipolar oscillation in a DEER measurement. To detect a longer coherence time *T*_2_ and thereby increase the maximum DEER distance *r*_max_, a dynamical decoupling sequence (a train of densely spaced π-pulses)^31,32^ was applied instead of a single π-pulse in Fig. 2. The longer coherence time under dynamical decoupling *T*_2,DD_ leads to an improved detection scheme for DEER while measuring identical DND samples. Thereby, the inter-pulse delays *τ* were kept constant to avoid “spin echo modulations”^33^ and the total spin evolution time was prolonged by adding time blocks of (*τ*–π–*τ*)_*n*_ (with *n* being an even number). Using such a dynamical decoupling sequence (taking the pulse phase alteration of the XY8 scheme^31,34^) on a single channel (Fig. 2 without MW2), a prolonged coherence time of *T*_2,DD_ = 21 μs was achieved (Fig. 3(b)). To the best of our knowledge, this is the longest coherence time of a single NV^−^ center in a 5 nm DND and represents a ten-fold increase of the coherence time compared to *T*_2_ = 2.1 μs for the same NV^−^ center (Fig. 3(a)). The corresponding estimated maximum accessible distance in a DEER experiment is *r*_max_ ≈ 11 nm. This suggests that the dipole–dipole coupling between two adjacent 5 nm DND, each containing an NV^−^ center, should be in reach.

An ODMR signal from two NV^−^ centers within a confocal spot still leaves possible distances of more than 200 nm, where there is no chance to measure a dipole–dipole oscillation from a DEER experiment. To narrow down the distance range, we preselected close DNDs containing NV^−^ centers using ODMR-based DESM super-resolution technique, which can be implemented on the same confocal ODMR microscope.^20,21^ On our confocal ODMR setup, a resolution down to 10–20 nm was achieved using ODMR-based DESM. The optimized experimental protocol, including the preselection of ODMR-based DESM, before running a final DEER experiment was the following:

(1) Recording continuous-wave (CW) ODMR spectra:

(a). Observation of spectrally separated four transitions from two NV^−^ centers with different orientations (see Fig. 1(b)).

(b). Detection of high ODMR contrast (at least for one of the two NV^−^ centers).

(2) Recording super-resolution images of NV^−^ centers in DNDs using ODMR-based DESM:^20^

(a). Selection of closest pairs of NV^−^ centers (and their host DNDs), which are within the spatial resolution limit of DESM (*ca.* 10–20 nm).

(3) Recording *T*_2_ measurements using a spin echo or dynamical decoupling sequence:

(a). Achievement of a long coherence time *T*_2_ (under a simple Hahn echo, see Fig. 3(c) and *T*_2,DD_ (under a dynamical decoupling sequence, here XY8,^34^ see Fig. 3(d))).

The DEER signals were directly compared to a single MW-frequency Hahn echo with π-pulses only on MW channel 1 (only blue MW pulses in Fig. 2) to avoid artefacts.^15,35^ From all of the candidates that passed three stages of the pre-selection (*ca.* 10 NV^−^ DND pairs), none of them showed a dipolar oscillation in the DEER signal (with or without dynamical decoupling).

### STORM

**Fig. 4:**
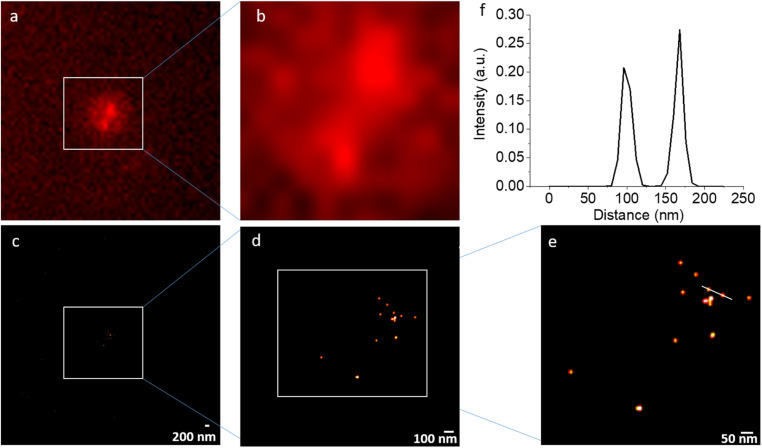
(a and b) A representative frame of the wide-field fluorescence (TIRF) images (raw data) and (c–e) the corresponding reconstructed super-resolved STORM images of aggregated 5 nm DNDs under continuous 561 nm excitation: images (a) and (c) as well as (b) and (d) show the same field of view. While the conventional fluorescence images cannot discriminate individual spots, the reconstructed images show several individual localizations of NV centers in DNDs. (f) A distance between two NV centers can be extracted: the white line in (e) leads to the distance distribution shown above. The localization precision cut off was at 15 nm.

NV STORM experiments have been carried out on a commercial wide-field fluorescence microscope dedicated for localization-based super-resolution imaging (Nikon N-STORM) in total internal reflection fluorescence (TIRF) mode. As a sample, non-irradiated DNDs that were surface-terminated with hydroxyl (OH) groups^21^ in Milli-Q water were applied as a drop on a quartz coverslip. The drying led to aggregation of DNDs (clusters of up to 1 μm) on the quartz cover slip. Fig. 4 shows the result of a representative STORM experiment under continuous laser excitation with *λ* = 561 nm, which matches the wavelength of the optical transition for NV^−^ centers, with an exposure time of 20 ms and 20 000 frames recorded. While individual NV spots cannot be resolved due to the diffraction limit in the conventional wide-field fluorescence images (Fig. 4(a) and (b)), the reconstructed super-resolved STORM images show many individual spots with a localization precision of less than 15 nm. The resolution achieved is better compared to that achievable with common organic dyes used in STORM imaging of biological samples due to the long duration of the blinking cycles. Despite the large aggregate of DNDs, the bright spots remain sparsely distributed. This is in agreement with an estimation from ensemble EPR measurements that only one out of 1000 DND particles contain an NV^−^ center, if particles are not electron irradiated to create further NV centers.^36^ Therefore, using the STORM approach, distances between DNDs containing NV centers from 15 nm up to several micrometers (only limited by the field of view (FOV)) can be determined within a precision of 15 nm.

**Fig. 5:**
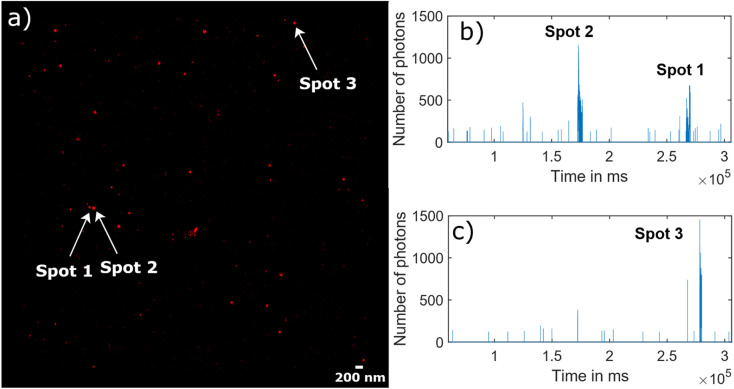
Blinking traces of NV centers in DNDs during a STORM acquisition. (a) Super-resolved reconstructed STORM image, where three bright spots corresponding to three NV centers are indicated with an arrow. (b) Blinking traces from the diffraction limited area of “Spot 1” and Spot 2′′, which are activated at a different time point of the time trace. (c) Similar blinking trace from the area of “Spot 3”.

Fig. 5 illustrates the raw data behind the super-resolved reconstructed STORM image (Fig. 5(a)). As an example, the blinking time traces in two different diffraction limited spots were extracted, where three super-resolved spots were reconstructed (Fig. 5(b) for “Spots 1&2” and Fig. 5(c) for “Spot 3”). The fluorescence signal appears as bursts during a short time of several tens of seconds rather than continuous on and off cycles over minutes.

## Discussion

While the STORM super-resolution imaging of NV centers in DNDs, based on the fluorescence blinking, led to successful localization with a precision down to 15 nm, the ODMR measurements of the DEER experiment could not detect a dipole–dipole coupling to an adjacent DND containing an NV^−^ center. Here, the simplicity of the STORM experiment outperforms the sophisticated DEER experiments: this is a purely optical experiment, which only relies on the fluorescence blinking of the NV center besides the optical excitation and detection, the DEER experiment needs a strong ODMR contrast (*ca.* 10% for DNDs in this work) and a long electron spin coherence time *T*_2_ of NV^−^ centers in DNDs. In the most favorable case the NV^−^ electron spin coherence time was *T*_2,DD_ = 21 μs using the dynamical decoupling (DD) sequence XY8. This makes a maximum distance in a DEER experiment of *r*_max_ ≈ 10 nm accessible. While this would be an excellent result for a spin label (a small molecule), the 5 nm size of the DND host crystal containing the NV^−^ center nullifies the advantage.

The localization precision of the two super-resolution imaging techniques STORM and ODMR-based DESM for NV^−^ centers in DNDs^21^ are comparable with 15–20 nm. The ODMR-based DESM method combines elements of both techniques: like STORM, it relies on super-resolution imaging technique and like DEER, it is based on the ODMR effect. The ODMR-based DESM method is technically simpler to implement than DEER experiment and, importantly, it does not depend on electron spin coherence time *T*_2_, since it is a CW (in contrast to pulsed) ODMR experiment. The selection criteria for the NV centers for the ODMR-based DESM method is less strict than for DEER. Compared to ODMR-based DESM, the STORM approach might be more accessible as an optical microscopy technique, since no additional magnetic and MW fields are needed and a commercial fluorescence microscope is sufficient as a setup. As an important advantage, STORM is a higher throughput method as it can record all the localizations of individual NV centers in a single measurement, while in ODMR-based DESM one has to repeat a measurement for each N–V orientation with a given MW frequency. One challenge of ODMR compared to pure optical methods is that only a fraction of the fluorescent photons (*i.e.*, the difference brightness between the electron spin states *m*_*S*_ = 0 and *m*_*S*_ = ±1),^6^ contributes to the signal-to-noise ratio. In our setup, for an NV^−^ center in a DND with 20 kcts s^−1^ and an optical contrast of 10%, the difference is about 2 kcts s^−1^.

In our STORM experiments, an unambiguous assignment of the dark state to the NV^0^ charge state^30^ cannot be made, since the photon intensity drops down to the noise level, rather than staying at a detectable lower intensity level given the overlapping emission spectra of NV^−^ and NV^0^.^29^ Whether or not the dark state is yet another charge state of the NV center remains to be determined.^18^

Due to the high number of paramagnetic defects, the coherence times *T*_2_ of NV^−^ centers in DNDs depend on the concentration of substitutional nitrogen defects *N*_S_ in diamonds (also called “P1 centers” in the EPR literature).^37^ Recently, we have estimated the concentration of P1 centers in DNDs to be around 1000 ppm.^38^ Using this value, the expected average coherence time *T*_2_ in DNDs would be around *T*_2_ ≈ 100 ns.^37^ A direct comparison is difficult due to several factors: (1) the P1 concentration dependence was carried out with ensembles of NV^−^ centers in bulk diamonds,^37^ (2) a concentration as high as [P1] = 1000 ppm was experimentally not covered in the given work^37^ and (3) the experimental difficulty to measure a fair average *T*_2_ value from single particle DNDs remains challenging, due to a bias towards the selection of the best NV^−^ centers.

A general challenge to use NV^−^ centers as labels for DNDs is that the majority of the nanodiamonds does not contain such a defect. However, we have shown that the NV^−^ concentration can be successfully enriched up to 1 NV^−^ center in 80 DND particles through electron irradiation.^38^ The NV^−^ concentration is only limited by the electron irradiation fluence and could be further improved through a longer irradiation time. There is a low probability that a pair of DNDs containing each an NV^−^ center is situated within a confocal spot, however, such a pair can be easily recognized by the two-fold fluorescence intensity from two individual NV^−^ signals (*ca.* 40 kcts s^−1^ instead of *ca.* 20 kcts s^−1^ in our case). For DEER experiments in DNDs, NV^−^ centers with a large ODMR contrast and a long electron spin coherence time *T*_2_ must be preselected before the experiment. This could be speeded up by an automated screening protocol.

Non-aggregated DND samples were prepared by spin coating aqueous solutions of fully dispersed DNDs^36^ on quartz microscope coverslips (see Fig. S7 in ESI‡ for AFM images). However, no fluorescence signal (continuous or blinking) from NV^−^ centers could be recorded and only one-time flashing spots were detected. Since fluorescent signals from optical defects in quartz coverslips cannot be excluded,^39^ an assignment based on optical lifetime measurements or photoluminescence spectra remained impossible due to the small number of photons collected.

Currently, the limit to use DNDs containing NV^−^ centers as distance labels is the rapid bleaching in their deaggregated states. Experiments were repeated with non-aggregated DNDs that were covalently bound to hyperbranched polyglycerol (HPG)^40,41^ to coat the particles with a thin polymer layer. This approach was inspired by promising results, where isolated single-digit HPHT nanodiamonds^42^ and DNDs^9^ showed stable fluorescent signals from embedded NV^−^ centers, after having been spin-coated with a layer of polyvinyl alcohol (PVA). However, HPG coating of DNDs did not improve the fluorescence stability of deaggregated DNDs. Since the chemical structures of the two polymers (HPG and PVA) are very similar, we assume that the thickness of the polyglycerol layer of a few nanometers on our DNDs^41^ was not enough to prevent photoionization to electron acceptor sites in the quartz substrate.^43^

## Experimental

### Materials and methods

#### DNDs

##### Preparation of DND samples

All 5 nm DNDs were obtained from Prof. Eiji Ōsawa (NanoCarbon Research Institute, Ueda, Japan). DND-OH (without electron irradiation) were prepared as described in ref. 21. Electron irradiated DNDs with a fluence of 10^19^ e^−^ cm^−2^ were prepared as in ref. 38 and fully deaggregated to a stable size of 5 nm in water (confirmed with DLS) using a boiling acid treatment.^36^ The concentration of DNDs containing NV-centers was estimated based on the half-field transition of the NV^-^ CW EPR spectrum (bulk measurement).^36,44^. The estimated defect concentration in non-irradiated DNDs is roughly 1 NV^−^ center in 1000 DNDs and increases to approximately 1 NV^−^ center in 100 DNDs after electron irradiation with a fluence of 10^19^ e^−^ cm^−2^.^38^

##### Preparation of microscope coverslips

To reduce the background fluorescence, quartz coverslips CFQ-2559, #No 1.5, 25 mm × 25 mm × 0.17 mm (UQG Optics) were used for the experiments. For cleaning, the cover slips were sonicated for 15 min. in acetone, sonicated for 15 min. in isopropyl alcohol, dried with nitrogen gas and finally applied to oxygen plasma for 15 min. to remove organic material. Atomic force microscopy (AFM) images of DNDs on quartz microscope cover slips were recorded on a FastScan AFM (Bruker).

#### DEER

##### ODMR/DEER microscope setup

NV^−^ DEER experiments in DNDs were carried out on a home-built confocal fluorescence microscope dedicated to ODMR spectroscopy.^45^ Two independent microwave channels enabled excitation at the two distinct DEER frequencies, where a four-channel arbitrary waveform generator (spectrum generator netbox dn2.66 × 04, Spectrum Instrumentation, Germany) supplied the two complex waveforms for up-conversion of each channel. To increase the chance of two close pairs of DNDs containing NV^−^ centers, aggregates of 10^19^ e^−^ cm^−2^ electron irradiated DNDs^38^ after boiling acid treatment^36^ were studied on a quartz microscope coverslip. Electron irradiation creates vacancies in the diamond lattice to enhance the NV^−^ concentration. The estimated defect concentration based on EPR spectroscopy after 10^19^ e^−^ cm^−2^ irradiation is roughly 1 NV^−^ center in 100 DNDs and therefore one order of magnitude larger than in the non-irradiated sample.^38^ A single NV^−^ in a DND had approximately a brightness of 20 kcts s^−1^ using an avalanche photo diode (PerkinElmer, SPCM-AQRH-16-FC 20754) in our setup. To identify spots with two NV^−^ centers within a confocal area, isolated fluorescent confocal spots with a brightness of around 40 kcts s^−1^ were selected.

##### Setup of DEER experiments

To initially identify NV^−^ centers, confocal scans without a magnetic field were recorded and CW ODMR spectra with a dip at *D* = 2.87 GHz were selected. After attaching a magnet, CW ODMR spectra were repeated and spots with spectrally distinguished four lines (corresponding to two NV- centers with a different orientation with respect to the external magnetic field) were selected. Pulse experiments were carried out at the resonance with the strongest ODMR contrast (NV1). All experiments recorded “upper” and “lower” traces, where for the latter, an adiabatic inversion pulse^46^ inverted the electron spin state from *m*_S_ = 0 (bright fluorescence) to *m*_S_ = +1 or *m*_S_ = −1 (darker fluorescence) right before the pulse sequence. The difference of the “upper” and “lower” trace led to the final trace. A Rabi nutation was performed to determine the lengths for π/2- and π-pulses.^21^ Then, a Hahn echo was performed by incrementing the delays *τ* and the coherence time *T*_2_ was extracted from a stretched exponential fit of the decay curve.^22^ The same procedure was repeated on an ODMR transition of the second NV^−^ center in the confocal spot (NV2). For the DEER experiment, the π-pulse on the second MW channel was centered around the π-pulse of the first MW channel. The DEER experiment was recorded by incrementing the delays *τ*. As a control experiment the simple Hahn echo (pulsing only on NV1) was recorded at the same time. This was done to exclude modulations of the Hahn echo caused by hyperfine couplings,^15,35^ which have to be distinguished from the mutual dipole–dipole coupling between NV1–NV2. For dynamical decoupling experiments, the single π-pulse in the Hahn echo was replaced by a train of equally spaced even-numbered π-pulses,^47^ where the pulse phases followed the XY8 scheme.^34^ A decay was recorded by adding blocks of even-numbered pulses, while keeping the inter-pulse delay *τ* constant, and the coherence time under dynamical decoupling *T*_2,DD_ was extracted from a stretched exponential fit of the decay curve.^22^ Similarly, the DD-DEER sequence was set up and the π-pulse on the second MW channel was centered around the π-pulse of the first MW channel.

#### STORM

##### Single-molecule localization and image analysis

Nanodiamonds were imaged using a Nikon N-STORM microscope (Nikon, UK Ltd) using an SR Apochromat TIRF 100 × 1.49 N. A., oil immersion objective lens. The illumination powers of light sources are reported as measured at the tip of the optical fiber. Fluorescence was detected with either an Orca Flash 4 v3 (Hamamatsu) or an EM-CCD Camera iXon DU897 (Andor). Imaging was performed in total internal reflection (TIRF) illumination mode to image close to the region above the coverslip. The field of view imaged typically covered 128 × 128 camera pixels corresponding to an area on the sample of ∼20 × 20 μm^2^. An in-built focus-lock system was used to prevent axial drift of the sample during data acquisition. The emission was collected and passed through a Laser QUAD filter set for TIRF applications, a multi edge dichroic filter with windows at 502–538 nm and 660–780 nm.

The laser excitation was at 561 nm, at a peak power density of 1.2 kW cm^−2^ and with an exposure time in the range of 10–30 m s.

##### STORM reconstruction

From each image stack, a reconstructed super-resolved image was generated by using the open source ThunderSTORM software, plugin of Fiji^48^ (ImageJ). Signals were detected searching the local intensity maxima in each frame, which were fitted using an integrated Gaussian point spread function. Localizations with an uncertainty above 15 nm and a *σ* (FWHM) above 250 nm were filtered out. Cross correlation drift correction was applied.

##### Extraction of blinking traces

Counts detected on the EM-CCD camera were converted to number of photons *N*_photons_ using the equation
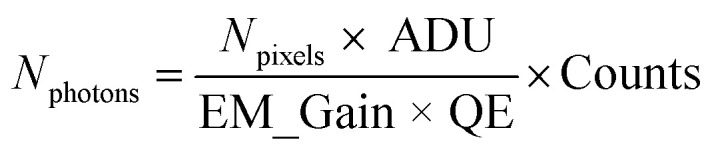
where *N*_pixels_ = 9 are the number of pixels, ADU = 5.19 is the analogue to digital unit (conversion factor to electrons), EM_gain = 300 is the gain factor and QE = 0.95 is the quantum efficiency of the EM-CCD camera at the emission wavelength.^49^ Blinking time traces from NV centers in DNDs were extracted in the software Fiji^48^ using the plugin “Time Series Analyzer V3”^50^ (with setting: “Get Average”). The first minute of the time traces was discarded to cut off bleaching of the background fluorescence. The remaining background (four times the standard deviation)^51^ was subtracted. Blinking time traces were background-corrected and the photon counts were summed over a ROI of 3 × 3 pixels.

## Conclusions

In this paper, two complementary methods to measure nanoscale distances between 5 nm DNDs including NV centers were evaluated. For the magnetic resonance pulse sequence DEER, the NV^−^ electron spin coherence time *T*_2_ was prolonged in a first step by a factor of ten to *T*_2,DD_ = 21 μs in the most favorable case using the dynamical decoupling (DD) sequence XY8. This improves the so far longest reported electron spin coherence time *T*_2_ = 4 μs of an NV^−^ center in DNDs^21^ by a factor of five. In units of the maximum accessible distance *r*_max_ in a DEER experiment, this increases *r*_max_ ≈ 6 nm to *r*_max_ ≈ 11 nm. With that, the distance measurement between two close DNDs containing an NV center, for example attached to different biomolecules, becomes in reach. The long coherence time *T*_2,DD_ is a key parameter for detecting oscillating magnetic fields^52^ and our findings improve the AC frequency range of 5 nanometer DNDs as quantum sensors. An increase of the MW amplitude will lead to shorter π-pulses, which would allow to place a higher number of refocusing pulses into the dynamical decoupling sequence. This experimental modification is expected to further prolong the electron spin coherence time *T*_2,DD_ of NV^−^ centers in DNDs.^53^ Despite the prolonged electron spin coherence time *T*_2,DD_ under dynamical decoupling, a mutual dipolar coupling between NV^−^ centers in aggregated DNDs could not be experimentally measured using DEER, probably since a close enough pair of nanodiamonds containing NV^−^ centers with a favorable orientation was not found. In contrast, using the photo-switching (“blinking”) approach of the super-resolution STORM experiment, nanoscale distances between individual DNDs were successfully reconstructed for aggregated DND samples on a microscope coverslip. STORM was applied for the first time to measure distances between different nanodiamonds, where previous measurements on NV centers had focused either on localization of several defects within a single diamond crystal^12^ or larger NDs.^18^ The localization precision down to 15 nm is comparable to the super-resolution images from ODMR-based DESM method in DNDs,^20,21^ but can be carried out on a commercial wide-field fluorescence microscope without the need for a magnetic field and a microwave setup (see Table 1).

## Author contributions

Conceptualization of NV-DND DEER experiment: TFS, RI and MS; Conceptualization of NV-DND STORM experiments: DP and TFS; funding acquisition: TFS, CD, MS, RI; acquisition and processing of NV/DEER experiments: TFS, RT, PA, KM and JB; acquisition and processing of STORM experiments: DP and TFS; chemical reparation of DND samples: FT-KS and DT under supervision of RI and MS; DND sample preparation for microscopy: KC; resources and supervision of ODMR lab: CD; software for ODMR microscopy: PW and JB; writing – original draft: TFS and DP; writing – review and editing: TFS, DP and CD.

## Conflicts of interest

There are no conflicts to declare.

## Supplementary Material

NA-005-D2NA00815G-s001
