# Supplementary material for: Distance measurements between 5 nanometer diamonds – single particle magnetic resonance or optical super-resolution imaging?
Source: Nanoscale Adv. 2023 Jan 24;5(5):1345–55. doi: 10.1039/d2na00815g (PMC9972529; doi:10.1039/d2na00815g)
Supplement: NA-005-D2NA00815G-s001 [file NA-005-D2NA00815G-s001.pdf]

Electronic Supplementary Material (ESI) for Nanoscale Advances.
This journal is © The Royal Society of Chemistry 2023

Electronic Supplementary Information

Nanoscale Advances

Distance measurements between 5-nanometer diamonds – Single particle magnetic resonance or optical super-resolution imaging?

Dorothea Pinotsi,[a] Rui Tian,[b,c] Pratyush Anand,[b] Koichiro Miyanishi,[d,e] Jens M. Boss,[b,f] Kevin Chang,[b] Pol Welter,[b] Frederick T.-K. So,[g,h,i] Daiki Terada,[g,h] Ryuji Igarashi,[i] Masahiro Shirakawa,[g,h] Christian L. Degen[b] and Takuya F. Segawa*[b,j]

a. Scientific Center for Optical and Electron Microscopy, ETH Zurich, 8093 Zürich, Switzerland
b. Laboratory for Solid State Physics, ETH Zurich, 8093 Zürich, Switzerland.
c. Current address: High-Field MR Center, Max Planck Institute for Biological Cybernetics, Tübingen, Germany
d. Graduate School of Engineering Science, Osaka University, Toyonaka, Osaka 560-8531, Japan
e. Center for Quantum Information and Quantum Biology, Osaka University, Osaka 560-8531, Japan
f. Current address: Neurocritical Care Unit, Department of Neurosurgery and Institute of Intensive Care Medicine, University Hospital Zurich, 8091 Zürich, Switzerland
g. Department of Molecular Engineering, Graduate School of Engineering, Kyoto University, Nishikyo-Ku, Kyoto 615-8510, Japan
h. Institute for Quantum Life Science, National Institutes for Quantum Science and Technology, Anagawa 4-9-1, Inage-Ku, Chiba 263-8555, Japan
i. Institute of Chemical Research, Kyoto University, Uji, Kyoto 610-0011, Japan
j. Laboratory of Physical Chemistry, ETH Zurich, 8093 Zürich, Switzerland, E-mail: segawat@ethz.ch

Protocol: ODMR spectroscopy of NV^- centers in DNDs

1. Isolated fluorescent spots were identified by recording a confocal scan (Fig. S1).
2. An continuous-wave (CW) ODMR spectrum is recorded at such a fluorescent spot (Fig. S2) using a weak (< 10 mT) external magnetic field. If four spectrally distinguished ODMR peaks are identified (corresponding to two NV^- centers in two different DNDs), the spot is selected for further measurements.
3. At one of the ODMR resonance frequencies (belong to NV1), an ODMR Rabi oscillation is recorded to determine the pulse lengths for $\pi/2$ and π-pulses (Fig. S3).
4. A Hahn echo is recorded with the calibrated pulse lengths to determine the coherence time T_2 (Fig. S4).
5. A prolonged coherence time under a dynamical decoupling sequence is recorded to determine $T_{2,DD}$ (Fig. S5).
6. For DEER experiment, points 3-5 are repeated for an ODMR resonance of the second NV^- center (NV2).

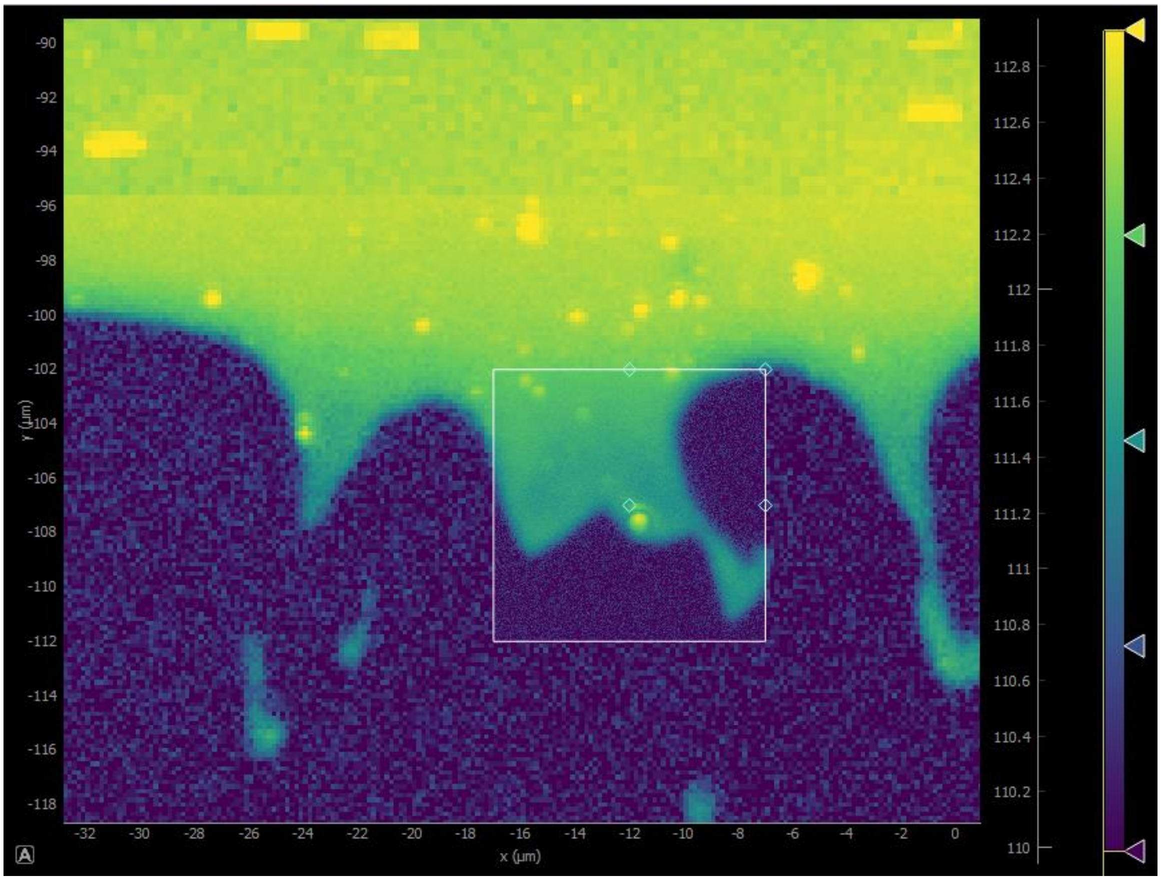

Figure S1. Confocal fluorescence image of drop-casted DNDs on a quartz coverslip. The studied NV^- center/DND is in the center of the white square.

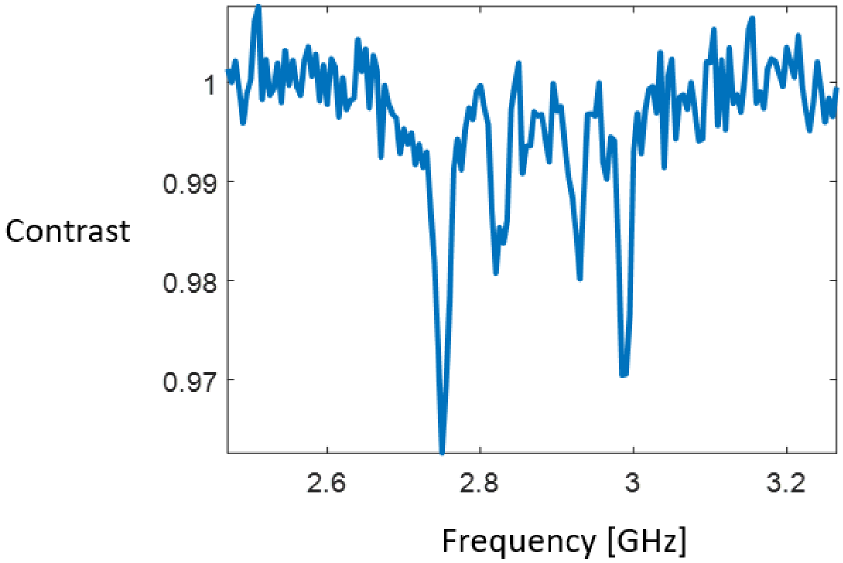

Figure S2. A CW ODMR spectrum recorded at the centered fluorescent spot in Fig. S2.

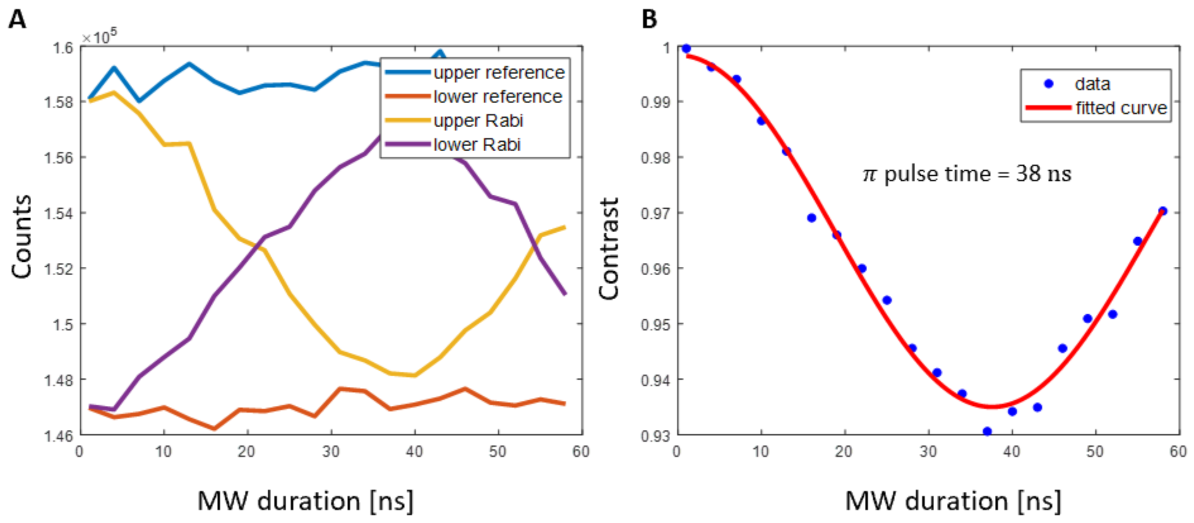

Figure S3. An ODMR Rabi oscillation recorded at the NV- ODMR resonance of 2.75 GHz (left most peak in ODMR spectrum of Fig. S3). The plots show the fluorescence intensity as a function of the Rabi MW pulse length in ns. A): Raw data with four traces: "upper reference": laser polarization pulse, "lower reference": laser polarization pulse followed by microwave (MW) adiabatic inversion pulse, "upper Rabi": laser polarization pulse, followed by MW Rabi pulse, "lower Rabi": laser polarization pulse, followed by MW adiabatic inversion pulse and Rabi pulse. B) Average Rabi oscillation is calculated from the raw data traces by $0.5 \times \left[1 - \frac{(Upper\ Ref - Upper\ Rabi)}{Upper\ Ref} + 1 - \frac{(Lower\ Rabi - Lower\ Ref)}{Lower\ Ref}\right]$. A π-pulse length of 38 ns was obtained, by fitting a exponentially decaying cosine modulation.

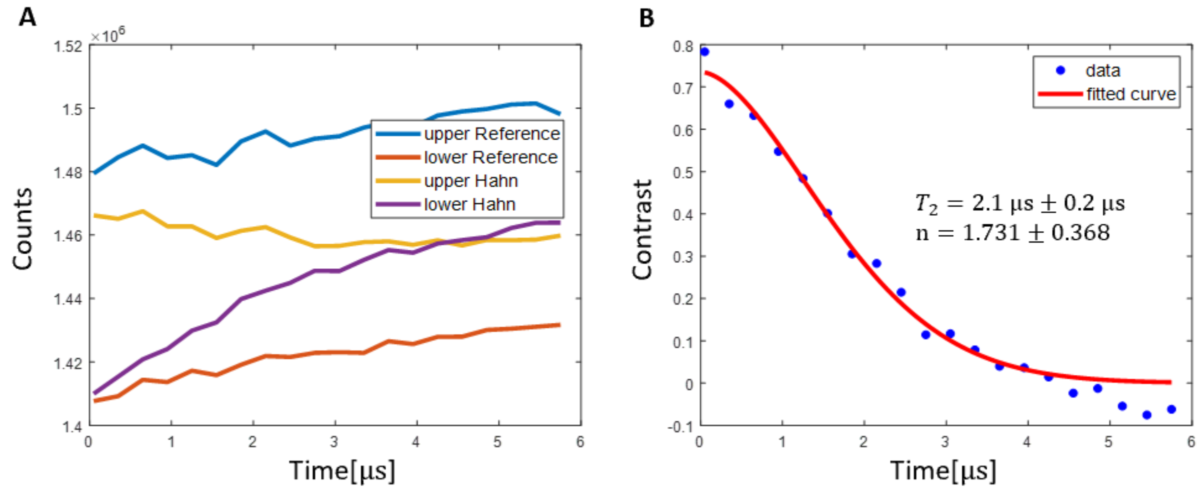

Figure S4. An ODMR Hahn echo (single π-pulse) using the pulse length calibrated int Fig. S4, leading to a coherence time of $T_2 = 2.1 \pm 0.2$ μs, when fitted with a stretched exponential (exponent $n = 1.7 \pm 0.4$).

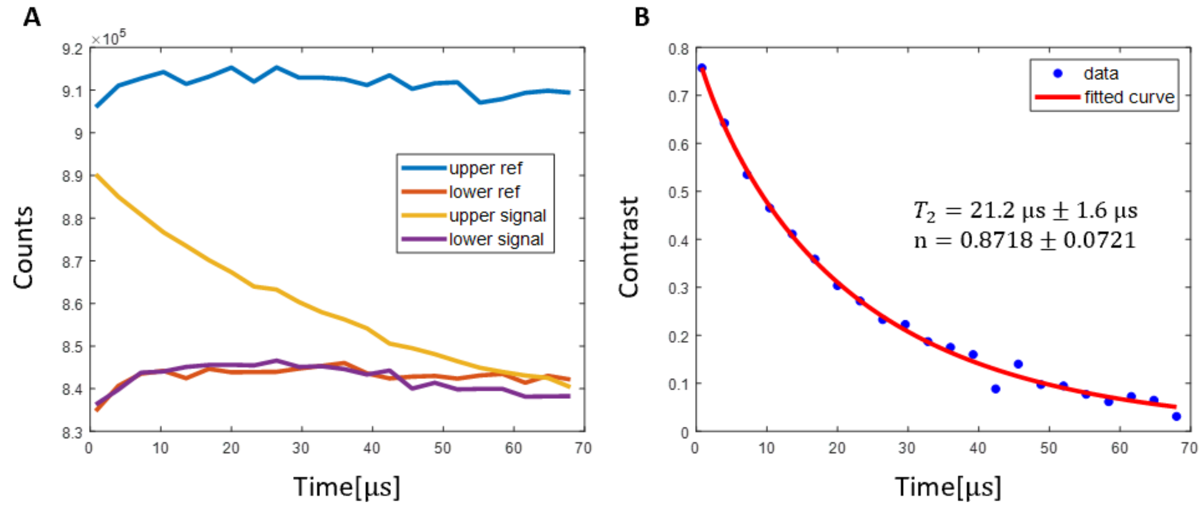

Figure S5. An ODMR signal under dynamical decoupling (train of π-pulses) using the XY8 phase scheme, leading to a coherence time of $T_{2,DD} = 21.2$ μs. The decay was fitted with a stretched exponential with an exponent $n = 0.9$.

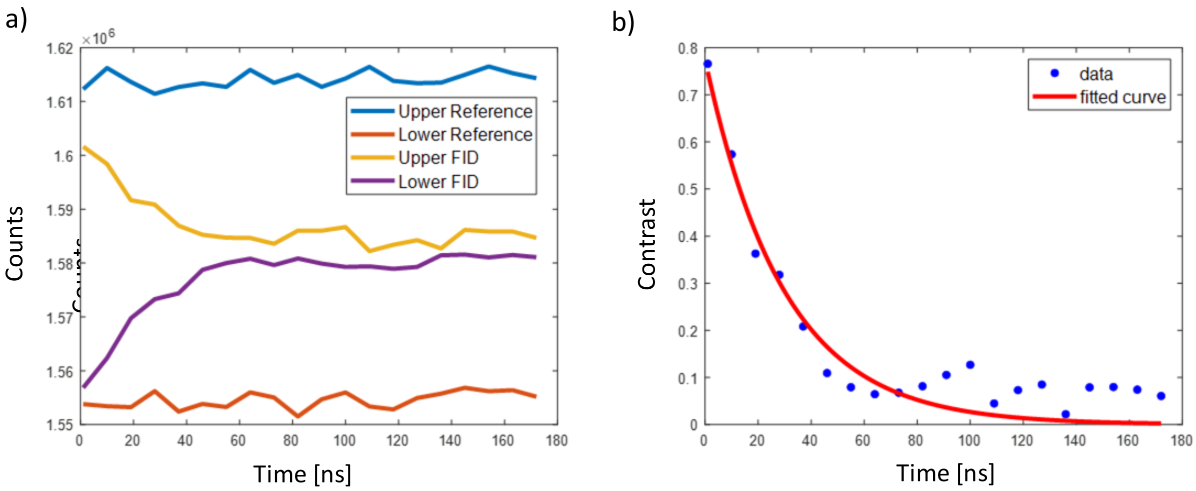

Figure S6.. For further characterization, the T_2* decay of a Ramsey signal (called “Free Induction Decay” in magnetic resonance) of the same NV^- center in DND was recorded. The mono-exponential fit led to of $T_2^* = 30 \pm 6$ ns.

AFM images of DND samples on quartz coverslips

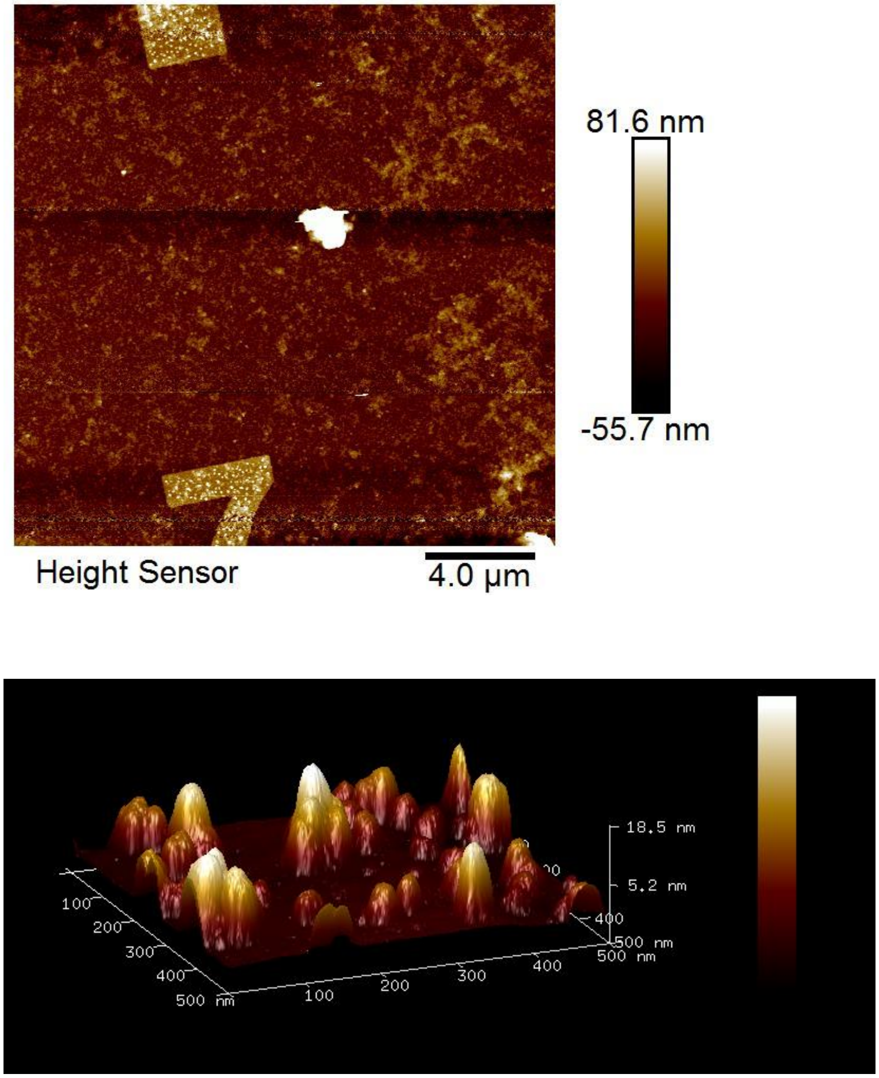

Figure S7. Above: AFM image of large DND aggregates of DND-OH (initial sample) after drop-casting and drying. Below: AFM image of dispersed DNDs (after boiling acid treatment), spin-coated onto the quartz coverslip. While the aggregated samples led to stable/blinking fluorescence of NV^- centers, the dispersed DND samples showed no fluorescence.
